# A Social Gradient in Fatal Opioids and Cocaine Related Overdoses?

**DOI:** 10.1371/journal.pone.0125568

**Published:** 2015-05-04

**Authors:** Alain Origer, Etienne Le Bihan, Michèle Baumann

**Affiliations:** 1 Drug Coordination Office, Ministry of Health, Luxembourg, Luxembourg; 2 Research Unit INSIDE, Institute Health & Behaviour, University of Luxembourg, Walferdange, Luxembourg; Brown University, UNITED STATES

## Abstract

**Background:**

To determine the existence of a social gradient in fatal overdose cases related to non-prescribed opioids and cocaine use, recorded in Luxembourg between 1994 and 2011.

**Methods:**

Overdose cases were individually matched with four controls in a nested case-control study design, according to sex, year of birth, drug administration route and duration of drug use. The study sample, composed of 272 cases and 1,056 controls, was stratified according to a *Social Inequality Accumulation Score* (*SIAS*), based on educational attainment, employment, income, financial situation of subjects and the professional status of their father or legal guardian. Least squares linear regression analysis on overdose mortality rates and *ridit* scores were applied to determine the *Relative Index of Inequality* (*RII*) of the study sample.

**Results:**

A negative linear relationship between the overdose mortality rate and the relative socioeconomic position was observed. We found a difference in mortality of 29.22 overdose deaths per 100 drug users in the lowest socioeconomic group compared to the most advantaged group. In terms of the *Relative Inequality Index*, the overdose mortality rate of opioid and cocaine users with lowest socioeconomic profiles was 9.88 times as high as that of their peers from the highest socioeconomic group (95% CI 6.49–13.26).

**Conclusions:**

Our findings suggest the existence of a marked social gradient in opioids and cocaine related overdose fatalities. Harm reduction services should integrate socially supportive offers, not only because of their general aim of social (re)integration but crucially in order to meet their most important objective, that is to reduce drug-related mortality.

## Introduction

Latest estimates from the United Nations Office on Drugs and Crime [1] and the World Health Organisation [2] refer to 210,000 and 245,000 drug-related deaths per year worldwide and approximately half of these cases are attributed to acute overdoses. Fatal overdoses caused by the use of illicit drugs in Europe account for an estimated 4% of premature death in young adults aged 15–39 years [3].

Social and economic conditions shape risk behaviors and the health of drug users as they impact on drug use patterns and affect the availability of resources and access to social welfare systems [4]. Nonetheless, fatal overdoses are avoidable to a large extend [5].

The existence of inequalities in health and mortality between groups with different socioeconomic profiles has been extensively documented since the 1980s [6–9].

Research focusing on social and economic determinants of drug-related mortality appears to be less prevalent, although previous studies have reported associations between fatal overdoses and individual parameters such as poor educational attainment [10–12], unemployment [13,14], low income [14,15], poverty status [15–17], homelessness [18,19] and poor psychosocial functioning [20].

Davoli *et al*. [21] investigated risk factors for drug overdose mortality, including 81 cases and 324 controls, matched on sex and year of birth and found no association with the educational status of victims. Galea *et al*. (2003) [22] applied a multi-level case-control study including 725 accidental overdose deaths and 453 accidental deaths due to other causes in various neighborhoods in New York City. Their findings showed increased odds of dying from overdose in neighborhoods with unequal income distribution as second level measure. In a further ecological study, including 59 residential community districts of New York City, Marzuk *et al*. [16] showed that the mortality rates due to overdoses involving cocaine and opiates are associated with poverty status. By comparing mortality rates according to causes and educational attainment in a cross-sectional study design, Borrell *et al*. [23] found highest relative risks of fatal drug overdose among victims with lowest educational profiles in the City of Barcelona. The study, however, included overdose deaths caused by both, illicit and prescription drugs.

The objective of our study is to investigating the existence of a social gradient in fatal drug overdose cases related to non-prescribed opioids’ and cocaine use by addressing the question whether the odds of a fatal overdose incident increase proportionally with the accumulation of social and economic disadvantages. Our findings should contribute to improving knowledge on the pathways that may lead to overdose incidents and to reduce their occurrence and consequences by implementing evidence-based public health, social and harm reduction policies.

## Methods

In the present study, a fatal overdose case is defined as an intentional or unintentional death for which an acute adverse reaction after the recent use of products containing non- prescription opioids and/or cocaine was reported as the primary cause of death according to toxicological and forensic evidence. Concomitant use of other substances is not an exclusion criterion, if the use of opioids and/or cocaine is reported as the primary cause of death by forensic authorities.

### Data sources

The following data sources have been linked and cross-examined in order to complete overdose victims’ profiles, drug use patterns and life histories:

#### Drug misuse surveillance data

The national drug monitoring system (RELIS) is operated by the Luxembourg focal point of the European Monitoring Centre on Drugs and Drug Addiction (EMCDDA) and indexes drug-related contacts with both, drug demand and drug supply reduction institutions in a single and integrated database. The RELIS network includes national psychiatric departments of general hospitals, specialised in- and outpatient drug care services, including opioid substitution treatment offers and harm reduction services, prisons and special drug law enforcement agencies.

Indexed drug users are digitally anonymised by means of an encryption algorithm approved by the National Commission on Data Protection. The derived attribution code allows respondents’ tracing within single and between multiple data sources while guarantying anonymity of subjects, as requested by national data protection authorities. The RELIS data protocol includes routine items on socio-demographics, educational, socioeconomic and health status, drug use patterns and histories, treatment records and contacts with the penal system.

#### Forensic evidence

In case of a suspicious death case, the public prosecutor’s office orders a toxicological investigation and an autopsy of the victim. The forensic department of the National Laboratory of Health (LNH) reports drug-related death cases also to the Ministry of Health for public health surveillance and statistical purposes. Forensic reports contain data on substances detected in the victims as well as an assessment of the association of detected substances and the occurrence of death. Autopsy reports also contain contextual information and elements of victims’ life history. Toxicological reports have been anonymised and made available to the research team by the LNH, following the authorisation of the Public Prosecutors Office.

#### National law enforcement records of fatal overdose cases

National judicial police authorities exhaustively list overdose deaths occurring on the national territory after forensic confirmation. Toxicological evidence as well as related police reports are compiled for each case.

Linkage of data sources was possible as RELIS codes have been calculated and attributed to all overdose victims, previously de-identified. Since persons included in the national RELIS database or in the Police overdose record are anonymised by the same coding routine, matching cases could be reliably detected.

### Matching procedures in the case-control setting

A nested case-control study design was applied. Cases are defined as victims of a fatal overdose having occurred in Luxembourg between 1994 and 2011. Matched controls refer to persons indexed by the epidemiological surveillance system on drug users in contact with national services (RELIS). Cases were matched with four controls for sex, year of birth, drug administration route and duration of drug use. Previous research has shown that matching more than four controls with each case does not significantly add statistical power to the analysis [24,25]. Controls were never matched to more than one case and deceased users were excluded from the RELIS database in order to avoid that they were matched with cases.

Matching variables are defined as follows:

- *Sex* (Male, female).
- Y*ear of birth* (In case no perfect match was available, a difference of one year more or less between the case and controls was accepted. Beyond age-specific risk differences, this variable also allowed to take account of potential changes in educational, economic and employment standards and contexts during the observation period).
- *Route of administration* (Injecting drug use, non-injecting drug use).
- *Duration of illicit drug use* (Matching according to duration of drug use is an essential condition to avoid the selection bias of selective survival. Risk exposure parameters of cases and controls were set to be comparable and in case no perfect match could be found, controls with the closest duration of drug use were chosen).

We used the *Relative Index of Inequality* (*RII*) to determine socioeconomic inequalities within the study sample, taking into account both, the size of sub-groups and the relative position of individuals within. In the present study, the *RII* measures the relative risk of a fatal overdose for the least advantaged group compared to the most advantaged group. The higher the *RII*, the more prevalent are socioeconomic inequalities within the study population.

The *RII* is a regression based index and only applies to linear changes in incidence (mortality) rates according to socioeconomic position parameters. To calculate the *RII*, we stratified our study sample according to a *Social Inequality Accumulation Score* (*SIAS*), based onfactors and values ^presented in Table 1. Scores attributed to each value have been weighted to ensure that the total weight of each factor is equal.

**Table 1:** Factors and values with associated weighted Social Inequality Accumulation Scores (*SIASs*).

Factors	Values	*SIAS*
*Educational attainment*	Primary/elementary school degree	0
	Lower secondary school degree	0.33
	Higher secondary school degree	0.66
	High school/university degree	1
*Paid professional activity/employment*	No	0
	Yes	1
*Main legal income*: *salary*	No	0
	Yes	1
*Financial situation*: *debt status* ^a^	Yes	0
	No	1
*Professional status of father or legal guardian*	Unqualified manual worker	0
	Qualified manual worker	0.2
	White collar, service provider	0.4
	Intermediate occupation	0.6
	Manager function (employed)	0.8
	Intellectual self-employed profession	1

^a^ Debt status: financial incapacity to cover ordinary life expenses or to reimburse debts (private debts, mortgage, loan, etc.) according to agreed or contractual terms and delays.

*SIASs* have been attributed to cases and to controls to determine their respective socioeconomic position within the sample. The lowest possible *SIAS* equals to 0, whereas the most beneficial socioeconomic position is quantified by a *SIAS* of 5. Knowing that the study population is not comparable to the national general population in terms of socioeconomic parameters [12], mortality (by overdose) could not be assessed in absolute terms but based on the relative socioeconomic position that each individual occupies within the distribution of socio-economic parameters of the study sample, composed of cocaine and/or opioids’ users only.

In order to determine the *RII*, the sample has been stratified into 4 groups according to *SIASs* situated within the following intervals: ≤1; >1 and ≤ 2; >2 and ≤ 3; >3. We calculated respective overdose mortality rates as well as relative frequency (RF) and cumulative frequency (CF) of survivors, both required to determine a parameter called *ridit* (r) [26] for each of the 4 *SIAS* groups the study population (cases and controls) is composed of, according to the following formula:

r=[CF+(CF−RF)]/2

Weighted least squares linear regression analysis on overdose mortality rates and *ridit* scores were applied and the *RII* was calculated according to the formula provided by Kunst and Mackenbach [27]. Sex and age were not applied as adjustment variables to determine the *RII* since they were used as matching variables in the case-control selection process.

In order to account for missing values, we performed multiple imputations using the fully conditional specification approach [28] on the entire data set and generated 10 imputation data sets. Results have been combined across the imputed sets of data according to the method recommended by Rubin [29] and the overall *RII* estimate is the average of the individual RII estimates. Statistical analysis was conducted using SPSS, version 21.0.

## Results

Our sample was composed of 1,328 persons including 272 fatal overdose cases and 1,056 controls. Six cases had to be excluded from the sample as no matching controls were found. For 3, 4 and 15 cases, respectively 1, 2 or 3 controls only could be matched. Eighty-one percent of controls matched cases for all matching variables, 4% were closest matches (+/- 1 year) for date of birth, 13% for duration of drug use career and 2% for both matching variables. The distribution of missing values for predictor variables is as follows: professional status of father or legal guardian (26.5%), debt status (16.8%), legal income, salary (13.7%), educational attainment (10.7%), and professional inactivity/unemployment (6.9%).

The overall characteristics of national fatal overdose victims and the case-control study sample were further described in previous publications [12,30].

Descriptive results (Table 2) reveal that FOD victims concentrate in lower *SAIS* classes. The mean *SIAS* of fatal overdose victims is 1.22, compared to 1.85 for survivors (*t = 25*.*089; p = *.*000*).

**Table 2:** Mean *SIAS* and distribution within *SIAS* classes according to gender and fatal overdose (FOD) status.

*SIAS*	FOD	NON-FOD	TOTAL
*Mean*	1.22	1.85	1.74
*Min*.	0	0	0
*Max*.	5	5	5
*S*.*E*.	1.06	1.09	1.11
***SIAS* class**	**FOD**	**NON-FOD**	**TOTAL**
*1*	50.7%	26.9%	31.2%
*2*	29.0%	30.9%	30.5%
*3*	13.9%	25.8%	23.7%
*4*	6.4%	16.4%	14.6%

Table 3 presents overdose mortality rates according to *SIAS* classes as well as associated *ridit* scores.

**Table 3:** Overdose mortality rates and *ridit* scores associated to *SIAS* classes.

*SIAS* class	Overdose mortality rate (OMR)	N survivors	Relative frequency (RF)	Cumulative frequency (FC)	Ridit (r)
≤ *1*	0.29288	283	0.31152648	0.31152648	0.15576
*>1 and* ≤ *2*	0.17074	325	0.305373832	0.616900312	0.46421
*>2 and* ≤ *3*	0.10550	272	0.236760125	0.853660436	0.73528
*>3*	0.07806	173	0.146339564	1	0.92683

**Fig 1:**
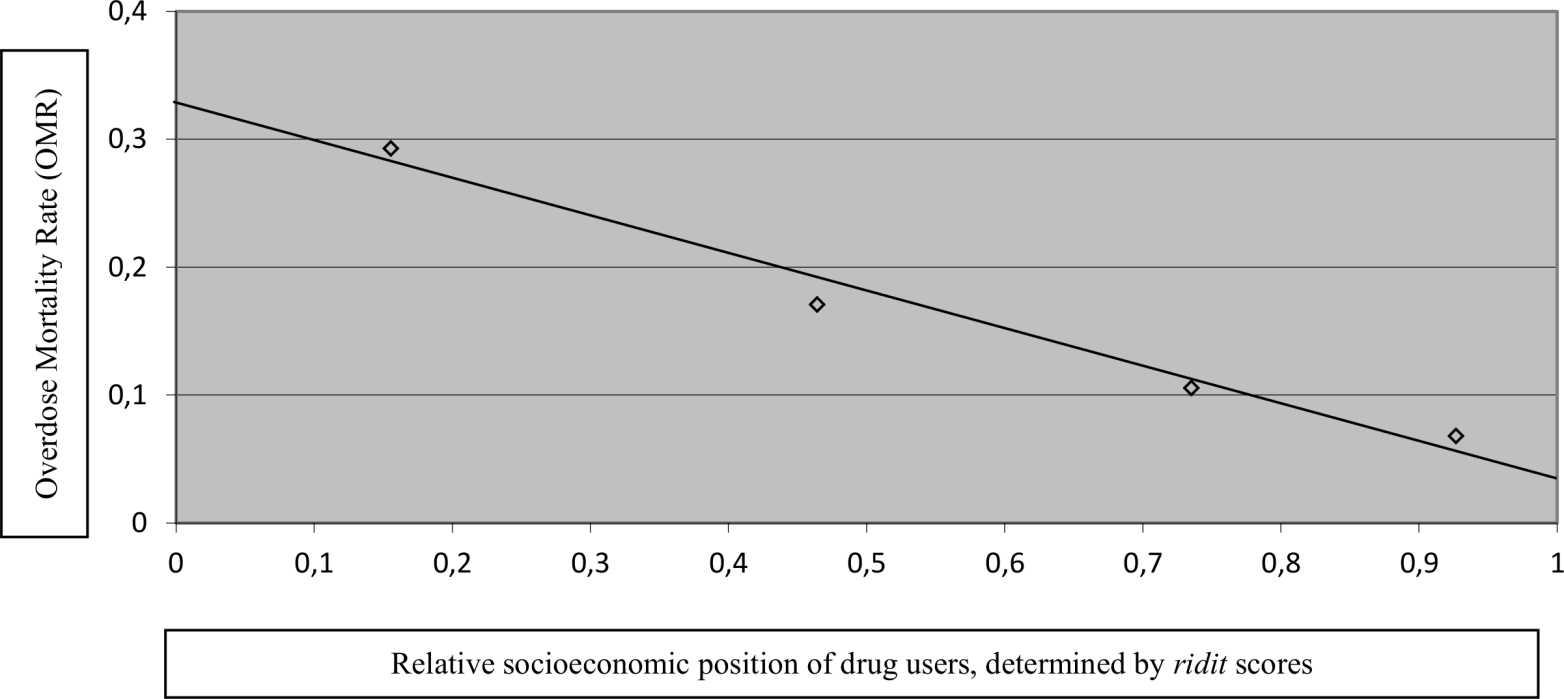
Overdose Mortality Rate (OMR) according to relative socioeconomic position determined by *ridit* scores associated to *SIAS*. Fig 1 shows that the overdose mortality rates decrease with increasing relative socioeconomic positions of opioids’ and/or cocaine users.

A negative linear relationship is observed between the overdose mortality rate and the relative socioeconomic position, determined by *ridit* scores, associated to *SIAS* (Fig 1).

We found an absolute difference in mortality of 29.22 overdose deaths per 100 drug users between the lowest *SIAS* class compared to the most advantaged class (Table 4). In terms of *Relative Inequality Index*, the overdose mortality rate of opioids’ and cocaine users with lowest socioeconomic profiles is 9.88 times as high [95% CI 6.49–13.26] as that of their peers from the highest socioeconomic group.

**Table 4:** Results of least squares linear regression analysis on imputed data, imputed *RIIs* and final *RII*.

Regression parameters	*S*.*E*.	*t*	Sig.	95% CI	*RII(s)*
***a*** ^a^ ***(1)*** 0.331	0.025	13.257	0.006	0.223 0.438	**11.41379**
***b*** ^b^ ***(1)*** -0.302	0.044	-6.916	0.02	-0.489–0.114	
***a (2)*** 0.325	0.031	10.48	0.009	0.192 0.459	**9.28571**
***b (2)*** -0.29	0.054	-5.353	0.033	-0.524–0.057	
***a (3)*** 0.334	0.029	11.382	0.088	0.208 0.46	**12.84615**
***b (3)*** -0.308	0.051	-6.004	0.027	-0.529–0.087	
***a (4)*** 0.326	0.024	13.775	0.005	0.224 0.428	**9.878788**
***b (4)*** 0.293	0.041	-7.07	0.019	-0.471–0.115	
***a (5)*** 0.328	0.032	10.245	0.009	0.19 0.466	**10.58065**
***b (5)*** -0.297	0.056	-5.298	0.034	-0.538–0.056	
***a (6)*** 0.328	0.03	10.907	0.008	0.198 0.457	**9.939394**
***b (6)*** -0.295	0.053	-5.626	0.03	-0.521–0.069	
***a (7)*** 0.317	0.012	26.594	0.001	0.266 0.369	**7.547619**
***b (7)*** -0.275	0.021	-13.171	0.006	-0.365–0.185	
***a (8)*** 0.327	0.025	13.274	0.006	0.221 0.433	**10.21875**
***b (8)*** -0.295	0.043	-6.834	0.021	-0.48–0.109	
***a (9)*** 0.317	0.006	50.243	0.000	0.29 0.344	**7.372093**
***b (9)*** -0.274	0.011	-24.853	0.002	-0.322–0.227	
***a (10)*** 0.327	0.033	9.946	0.01	0.185 0.468	**9.617647**
***b (10)*** -0.293	0.057	-5.11	0.036	-0.541–0.046	
***a* 0.326**					**9.88**
***b* -0.292**					**[95% CI 6.49–13.26]**

^a^ a: regression intercept

^b^ b: regression slope

In order to lay out the evolution of the *RII* and overdose mortality rates over time, a separate regression analysis was performed for two consecutive 9 years’ periods. Results showed a marked decrease in the *RII* from 17. 27 [95% CI 3.22–31.32] for 1994 to 2003 to 5.79 [95% CI 3.17–8.40] in reference to the period 2003 to 2011. Between the same time periods, a decrease in national overdose mortality from 6.37 to 4.22 cases^/100.000 inhabitants^ aged 15 to 64 has been observed.

## Discussion

### Key results

This study investigated whether the odds of dying from opioid and/or cocaine related overdose increase gradually down the socioeconomic positions users of these drugs occupy. Results suggest the existence of a marked social gradient in opioids and cocaine related overdose fatalities, extending beyond the mere binary difference in mortality between the most and the least advantaged on the socioeconomic scale. In other words, adverse socioeconomic conditions affect the overdose mortality risk according to a ‘*dose-response*’ scheme.

In terms of prevention and harm reduction strategies, this is a relevant finding as it suggests that any measure aiming at reducing social disparities, even if isolated or targeted, may have a positive and dynamic impact that counter the negative cumulative effect in terms of survival. This strongly supports the idea that drug-related harm reduction strategies exclusively focusing on reducing direct harm of drug use fail to address harm associated with the context of drug use such as homelessness, violence and poverty [31]. Also, harm reduction services should integrate socially supportive offers, not only to reach their general goal of social reintegration but crucially, because they can contribute to save lives, generate years of life without major impairments and have a sustainable positive impact on drug-related mortality, and thus meet their first and overall objective: reducing drug-related morbidity and mortality.

### Added value and generalizability

Studies on the association between single socioeconomic indicators and fatal overdoses in reference to the general population [14,32] and both, fatal and non-fatal overdoses in cross-sectional settings [33] have provided valuable outcomes in recent years. However, we could not identify previous case-control studies addressing inequalities in fatal overdose cases based on multiple socioeconomic parameters, combined into a cumulative measurement tool.

Moreover, the present study included probably for the first time, drug administration route and duration of drug use as matching variables in order to take due account of the risk exposition of both, cases and controls. These methodological aspects, as well as the large number of cases and controls and the cross-analysis of longitudinal data from different sources and settings, should add validity to our findings.

### Limitations and further research

The scarcity of studies on socioeconomic determinants of fatal drug overdoses may partly be due to the difficulty to gather data on social profiles of drug users. Parameters such as education, income, financial wealth, professional activity and socioeconomic situation of the family of origin are often not included in routine drug registration or monitoring systems.

Also, in a retrospective study design, poor flexibility is given as to the selection of study variables. Additional data on income sources of subjects and the socioeconomic status of their parents could have contributed to a more refined stratification of subjects according to their respective socioeconomic position.

Furthermore, research on drug users mostly rely on episodic data, covering the period from first or established drug use to the moment of data collection. In a life-course perspective, it would be beneficial to have access to early life and life trajectory data in order to allow a more in-depth analysis of cumulative effects of socioeconomic determinants. Therefore, a prospective cohort study design would have been most appropriate to address the present research topic, bearing in mind, however, the resources’ and time consuming nature of such studies.

We found that the *RII* has markedly decreased during the observation period along with a parallel reduction of the average overdose mortality rate. Future research should, beyond investigating the existence of socioeconomic inequalities in drug using populations, further address the association between the amplitude and distribution of these inequalities and mortality risks.

This said, social and economic parameters should not be seen as independent determinants and even more so should the concept of social inequalities be widened when applied to drug-related mortality. Whereas the social status seems to hold a major role here; family, social and societal environments, new communication means, mobility, migration and acculturation contexts as well as the emergence of new psychoactive substances, new consume patterns and related risks are at play in the attempt to explain differences in terms of morbidity and mortality associated to drug use. The challenge for future research will thus lie in the capacity to take into account the changing context and environments that next generations will experience and the way they will, or will not, succeed to sharing resources and distributing wealth in a ‘*healthy*’ way.
